# Analysis of subject specific grasping patterns

**DOI:** 10.1371/journal.pone.0234969

**Published:** 2020-07-08

**Authors:** Yair Herbst, Lihi Zelnik-Manor, Alon Wolf

**Affiliations:** 1 Faculty of Mechanical Engineering, Technion – Israel Institute of Technology, Haifa, Israel; 2 Faculty of Electrical Engineering, Technion – Israel Institute of Technology, Haifa, Israel; Indiana University Purdue University at Indianapolis, UNITED STATES

## Abstract

Existing haptic feedback devices are limited in their capabilities and are often cumbersome and heavy. In addition, these devices are generic and do not adapt to the users’ grasping behavior. Potentially, a human-oriented design process could generate an improved design. While current research done on human grasping was aimed at finding common properties within the research population, we investigated the dynamic patterns that make human grasping behavior distinct rather than generalized, i.e. subject specific. Experiments were conducted on 31 subjects who performed grasping tasks on five different objects. The kinematics and kinetics parameters were measured using a motion capture system and force sensors. The collected data was processed through a pipeline of dimensionality reduction and clustering algorithms. Using finger joint angles and reaction forces as our features, we were able to classify these tasks with over 95% success. In addition, we examined the effects of the objects’ mechanical properties on those patterns and the significance of the different features for the differentiation. Our results suggest that grasping patterns are, indeed, subject-specific; this, in turn, could suggest that a device capable of providing personalized feedback can improve the user experience and, in turn, increase the usability in different applications. This paper explores an undiscussed aspect of human dynamic patterns. Furthermore, the collected data offer a valuable dataset of human grasping behavior, containing 1083 grasp instances with both kinetics and kinematics data.

## Introduction

Virtual Reality (VR) and Augmented Reality (AR) are both rapidly developing technologies that are going to play an important role in numerous aspects of our lives. An example of such an application domain is the medical field, specifically, by improving minimal invasive surgery, medical training and rehabilitation through integrating haptic feedback with AR/VR [1, 2]. While the headsets providing visual and auditory feedback are being adopted widely in recent years, haptic feedback devices are still rarely seen outside of research labs. One of the reasons for this is the fact that haptic feedback devices are usually cumbersome and heavy, or provide a very basic level of feedback [3]. In addition, most of these devices are “generic” and do not adapt to the individual user. Many of the wearable haptic feedback devices operate from the inner part of the hand. These devices are connected between the different fingers or between the palm and the fingers and extract normal forces. Some are limited to rigid objects [4], while others are capable of emulating compliant objects [5]. Some of the devices include the addition of different modalities, such as vibration and shear forces, for example, as shown in [6]. A different approach for designing a haptic feedback device is exoskeleton devices that offer greater versatility in AR applications and a wider range of possible objects. However, these devices suffer from a lack of Degrees of Freedom (DoF) in comparison to the human hand, thus limiting their capabilities. These gloves range from one DoF per finger, as in the CyberGrasp (CyberGlove Systems LLC, CA, US), to more advanced devices based on different actuation techniques [7–9]. As mentioned, all of these devices are "generic" and could potentially benefit from the addition of personalization.

An entirely different class of devices, called encounter type devices, is also common. These devices are usually mounted on a table and are not wearable. Such a device is presented in [10]. The authors describe the process of observing human grasping behavior and how these observations were implemented in their design process. This type of design process can potentially improve design outcome.

Even though our over-reaching goal is to design a haptic feedback device, the scientific background relates to other fields, mainly grasping and hand biomechanics. These fields have been studied extensively with the research originating from various disciplines, such as physiology, occupational therapy and more. Much of the research is aimed at finding the grasping behavior among different subjects [11–15]. Methods for modelling the human hand skeletal system from surface measurements obtained from using motion capture systems with or without reaction forces also exist [16, 17]. The mechanical properties of the soft tissue surrounding the skeletal system also affect grasping greatly and are investigated in [18–21]. The topic of grasp synergies has also been studied to better understand the way human control and plan grasp tasks [22–24]. The topic of grasping of non-rigid objects presents a challenge of its own with haptics potentially playing a major role in the patterns [25, 26]. The conclusions from these types of studies are used for many applications, the most common one being robotic grasping, e.g. [27, 28]. These are examples of how scientific tools and prior knowledge of human grasping can be used in a bio-inspired design process. Such a design process has the potential of producing an improved design outcome.

Unlike the above-mentioned studies that were performed in order to find common properties within the population and thus gain a macro understanding of the behavior, we focused our study on the micro properties that make human grasping distinct rather than generalized. The best known example of subject specific motion patterns is biometric gait recognition [29]. Gait is considered a "soft" biometric method, meaning that it is not considered as distinctive as fingerprints, for example. Similarly, upper limb dynamics are also used for recognition [30, 31]. It is important to note that these distinguishable patterns can change over time. These examples might suggest that other aspects of our dynamic behavior are distinctive as well.

In this paper, we investigate differences in the dynamic patterns of human grasping. We hypothesize that subjects can be classified according to their biomechanical grasping parameters, i.e. kinematics and kinetics, and that these parameters would be dependent on the objects’ shape and properties, such as weight, stiffness and friction. Furthermore, we hypothesize that some of these features would be substantially more distinctive than others. To validate our hypothesis, we conducted experiments with 31 subjects who were asked to grasp five different objects. Our experimental setup is similar to previous research in the field of grasping (e.g. [14]) but the features and analysis were chosen with our end-goal of designing a haptic feedback device in mind.

Ten joint angles and five reaction force vectors were extracted from the collected data. The results of the research support our hypothesis. This paper discusses new aspects of human grasping patterns, we believe that the results reported could lead to a better designed process of haptic feedback devices, both mechanically and by optimizing the control loop, thereby improving the overall design outcome and the quality of the output feedback. Moreover, the dataset of kinematic and kinetics data collected during over 1000 grasp instances could help researchers better understand human grasping patterns, is by itself a major contribution.

## Methods

### Subjects

The research population includes 31 subjects who volunteered to participate in the experiments, 19 of whom are males and 12 are females (average age of 26). Of the 31 subjects, four were left-handed, similar to the ratio in the general population. None of the participants had a diagnosed orthopedic or neurological disorder of their hand. Written informed consent was obtained from each of the patients and approval was obtained before study commencement from the Institutional Review Board and Human Subjects Protection, US-HHS-FWA-00013345, The RB Rappaport Faculty of Medicine, Technion—Israel Institute of Technology, approval number: 21–2017.

### Measurement tools

#### Kinematics

**Fig 1:**
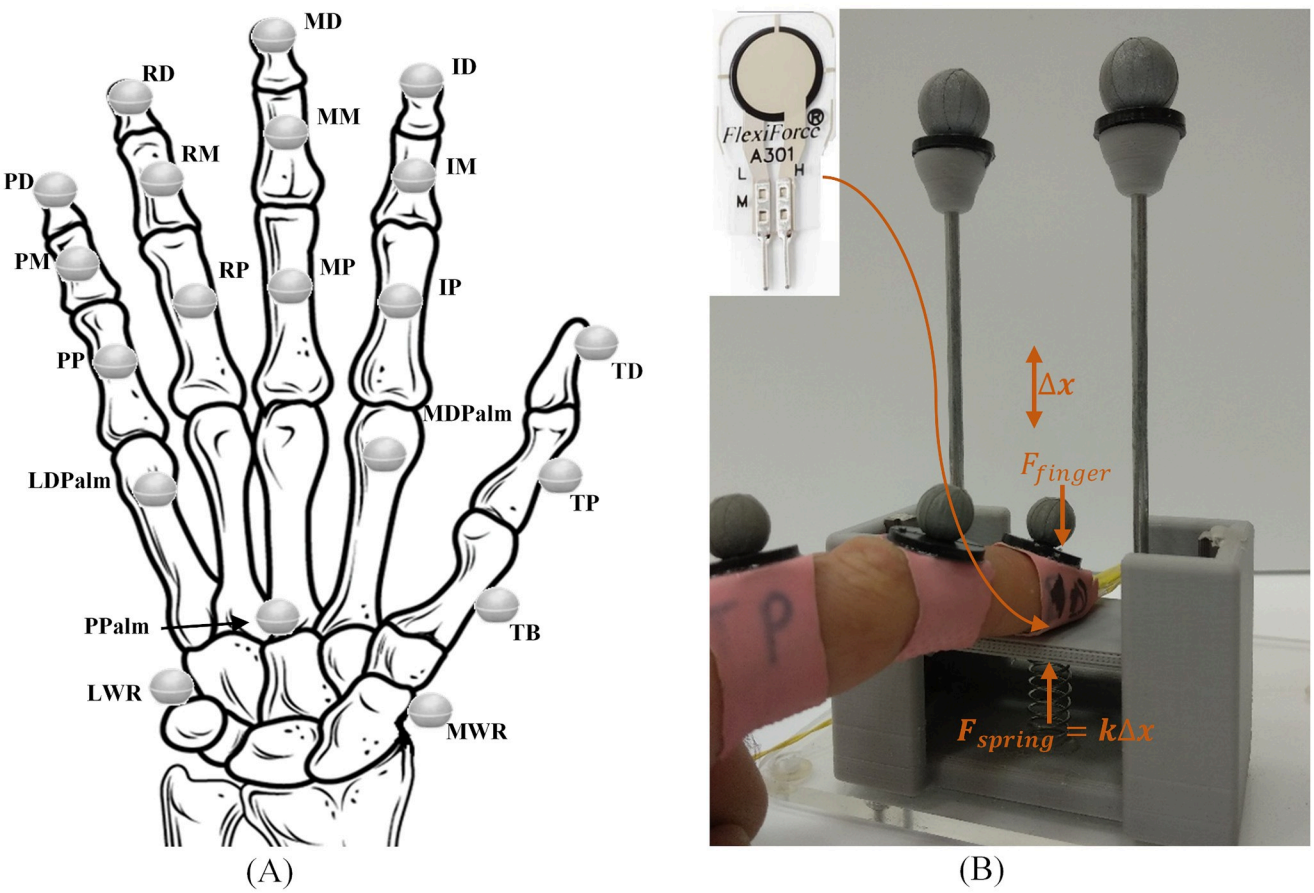
Measurement tools. (A) Reflective marker locations. (B) FlexiForce A301 sensor and its calibration mechanism. The user applies a force F on the platform through the sensor and a spring with a known constant resists the movement *F* = kΔ*x*, the movement of the platform is recorded using the Vicon reflectors mounted on the platform.

The hand kinematics was measured using a Vicon motion capture system (Oxford Metrics Ltd., Oxford, UK) due to its high accuracy (0.1mm). The system is comprised of 14 MX40 Vicon cameras collecting frames at 120Hz. To capture the hand movements, 20 9.5mm reflective markers were placed on the subjects’ dominant hand. As shown in Fig 1A, 15 markers are located on the middle of each of the phalanges, another three on the metacarpals representing the surface of the palm, and another two on the wrist joint, one on the scaphoid bone and one on the triquetral bone. The trajectories data collected by the Vicon were filtered with a 15Hz low pass filter to reduce noise.

#### Forces

Five FlexiForce A301 sensors (Tekscan Inc., Boston MA, US) were placed on the tips of the fingers to measure normal reaction forces with the object grasped. An evaluation of the quality of measurements using a glove-based device is presented in [32]. The analog data from the sensors was sampled at 960Hz through the Vicon MX Control and synchronized internally. The data was filtered using a bandstop filter to remove the 50Hz signal caused by the power grid. To minimize errors in the analog data, each subject calibrated the sensors before the measurement session. The calibration mechanism, shown in Fig 1B, is based on a moving platform mounted via two high precision miniature Mono Rail linear rails (Rollon S.p.A., Vimercate, MB, Italy) to a stationary base. The rails are connected in such a way that the platform is free to move up and down, parallel to the base of the device while maintaining very low frictional forces (*μ*_*s*_ = *μ*_*f*_ = 0.002 − 0.003). A compression spring (*k* = 0.45*N*/*mm*) was placed between the platform and the base. The subjects were asked to apply a downward force on the platform, one finger at a time, until the platform reached the end of the rail. This procedure was repeated three times to average the result and obtain a force-voltage calibration curve. Two reflective markers were mounted on the platform to track its position using the Vicon motion capture system.

### Experimental setup

**Fig 2:**
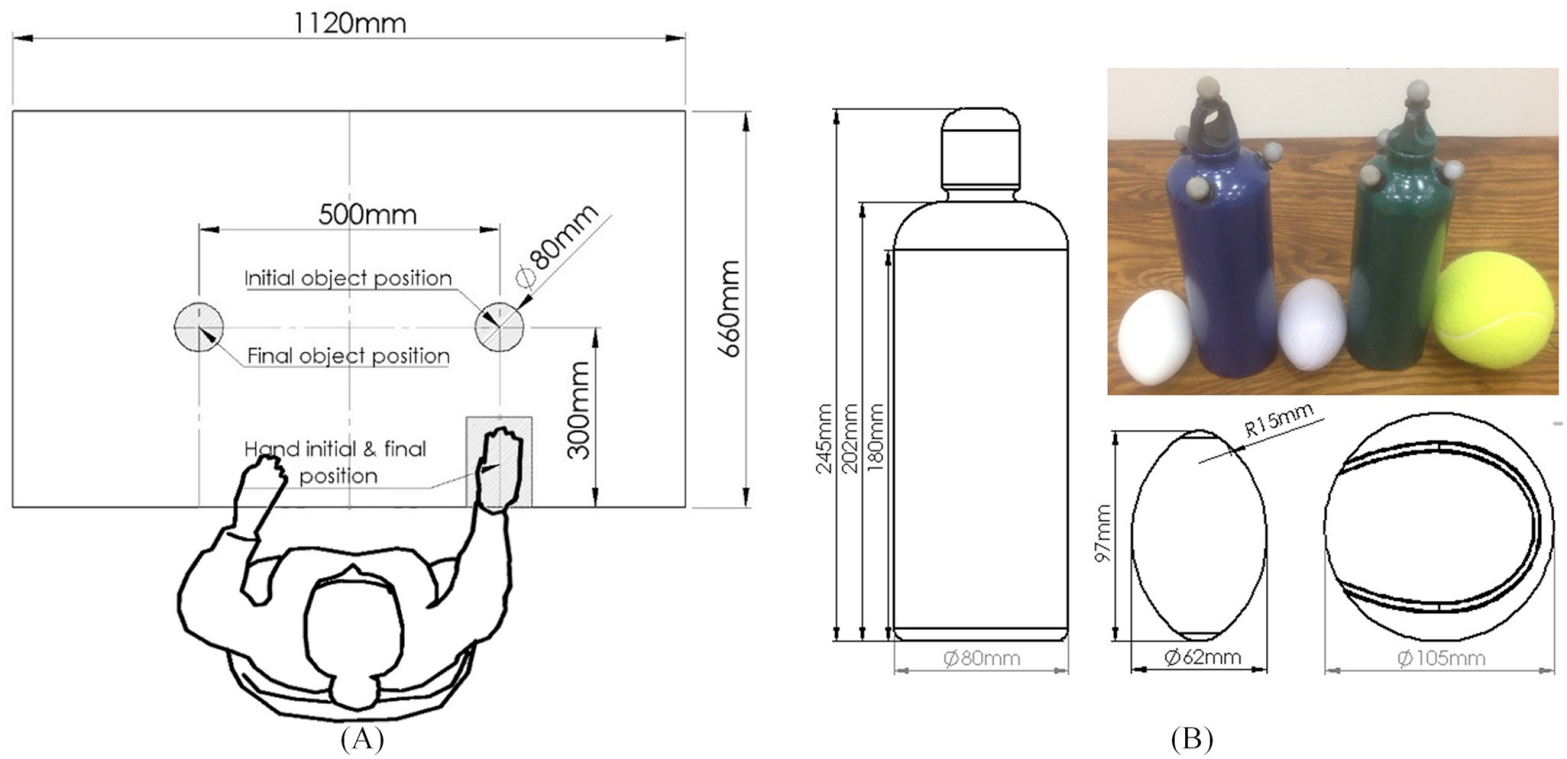
Experimental setup and objects. (A) The experimental setup. (B) The objects grasped during the experiment and their physical dimensions. From left to right: stiff 3D printed ellipsoid ball, full 1L water bottle, soft ellipsoid ball, half full 1L water and a tennis ball.

A detailed description of the experimental setup is given in Fig 2A. The subject was asked to sit in front of a table such that his/her sagittal plane was aligned with its center. The dominant hand rested pronated on the table 250mm from the center of the table and the wrist joint was aligned with the edge of the table. One of the objects was placed on the platform 300mm from the edge of the table on the same side as the hand. Then, the subject was asked to approach and grasp the object and move it to the symmetric (with respect to the centerline) point on the table. This task was repeated a total of seven times for each object.

The experiment was performed on five different objects. All the objects had at least one axis of symmetry, thus minimizing the effects of the orientation in relation to the subject. Overall, each subject performed 35 grasping tasks. Since the aim of this study was to find variability between subjects, the order of the grasping tasks remained constant between subjects, thus minimizing the effects on the results. The objects were as follows (shown in Fig 2B):

1. A tennis ball (105mm diameter)
2. Two identical one-liter aluminum water bottles (80mm diameter), one full and one half full, to examine the effects of weight variations
3. Two ellipsoid shaped balls, one soft (used for physical therapy) and one modeled to have the exact physical dimensions and 3D printed from stiff plastic. These two objects were used to investigate for stiffness and friction effects.

Smaller objects grasped using a pinch grip were avoided due to limitations of the measurement system. Moreover, since we were aiming to design a haptic feedback device in which small objects present their own challenges, we focused this study on larger, power/precision grasp objects.

### Feature extraction

Since our research originates from the intention of designing a haptic feedback device, we chose features relevant for this type of devices, joint angles and contact forces. While common feature spaces used in the analysis of grasping experiments include grip aperture, movement time, time to peak acceleration, etc. (e.g. [14]). These parameters do not seem to contain enough information for classification, since the shape of the kinematic measures is not compared [33]. For this reason, the entire time-dependent vector for each of the features was taken. Data analysis was carried out using Matlab software (Mathworks, Natick, MA, US). The recorded data was cropped from the instant that the hand started moving to the point it returned to its initial position, i.e. reach (movement commencement until contact with the object), grasp (contact with the object until object release) and release (object release until return to initial position). From the cropped data, 15 feature vectors were extracted.

#### Kinematics

**Fig 3:**
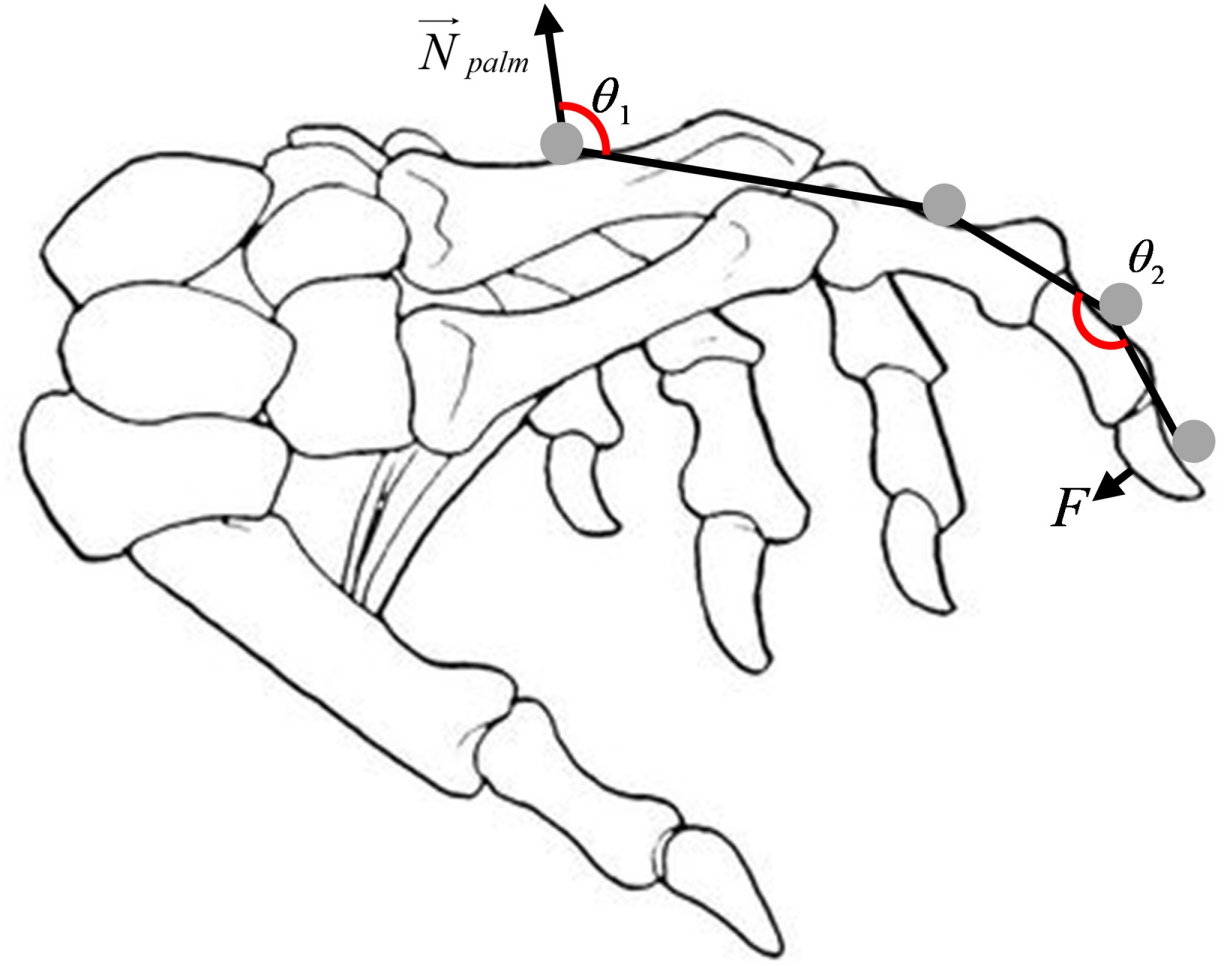
The three feature vectors representing each finger.

For fingers 2–5 (Index-Pinky), previous work has shown that most humans cannot control their distal and middle phalanges separately [34], meaning that, from a haptic feedback device perspective, one DoF suffices to provide feedback and thus to represent this movement. Moreover, since the abduction/adduction angles of the fingers are relatively negligible compared to the flexion/extension angles [34], these angles were not chosen as feature vectors. Bearing that in mind, two angles for each finger were calculated from the collected trajectories: *θ*_1_- the angle between the proximal phalange and the metacarpals and *θ*_2_- the angle representing the phalanges flexion (both illustrated in Fig 3). The angle between the proximal phalange and the palm was defined as the angle between the normal of the plane determined by the three palm markers (${\vec{N}}_{palm}$) and the line connecting the average point of these three markers and the marker on each one of the proximal phalanges. The angle representing the phalanges flexion was defined as the angle between the line connecting the proximal marker and the middle marker and the line connecting the middle marker and the distal marker.

While the Thumb performed movements that are much more complex than fingers 2–5, we chose only two feature vectors to represent its movement, thus providing a uniform feature space for all the fingers and giving a total of ten angles. The two angles for the thumb are: *θ*_1_- the angle between the thumb’s metacarpal and the palm and *θ*_2_- the angle representing the two phalanges flexion. The two angles were calculated similar to the other finger, where the marker on the proximal phalange was replaced by the marker placed on the Thumb’s metacarpals.

#### Forces

A total of five force vectors, one for each finger, were collected. The data was downsampled to 120 Hz to fit the data sampled by the motion capture system. Next, the voltage data collected was translated to force using the force-voltage curve that was obtained in the calibration step described earlier.

**Fig 4:**
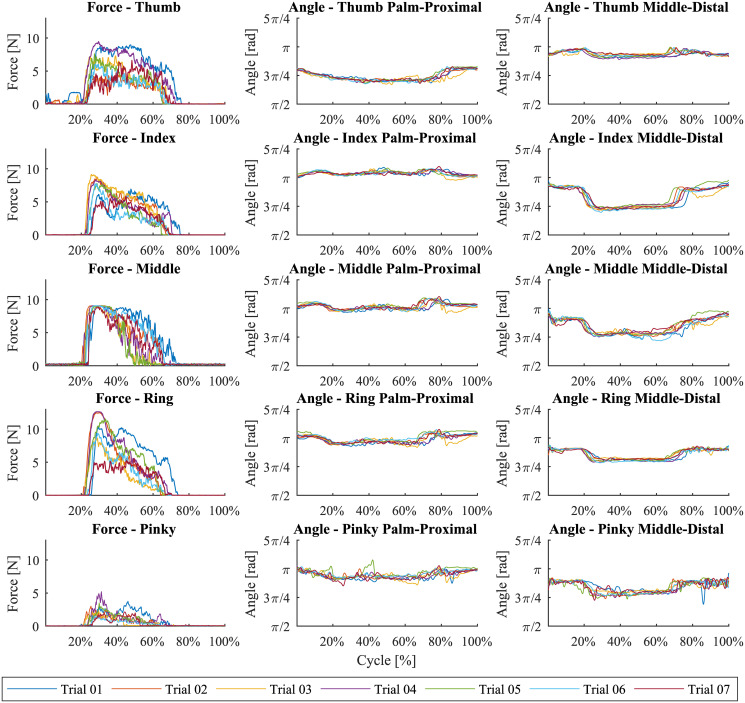
The extracted feature vectors for a specific subject and the half-full bottle. Each color represents one of the seven trials.

The extracted vectors were normalized in time to a constant length of 500 samples to eliminate the effects caused by the duration of the trial. The results for the half-full bottle and a specific subject are shown in Fig 4. It is important to note that, during non-contact phases (reach and release), the force vectors are strictly noise since the reaction forces are zero and, during grasp, the angles are relatively constant since there is no relative motion between the phalanges. The effects of the different phases and the corresponding features will be discussed in subsequent sections. After the feature extraction and rejection of trials with missing data points, we were left with 1083 trials divided between the five different objects. The first two trials for each object were considered as training trials and were not taken into account. The final dataset contained 774 trials, each of which was represented by a 15 features by 500 data points matrix.

### Data analysis

In order to test our hypothesis, the following methods were used:

#### Dimensionality reduction

A non-linear dimensionality reduction technique, called t-distributed Stochastic Neighbor Embedding (t-SNE) [35], was used to visualize and cluster the data. t-SNE is particularly suitable for reducing high-dimensional data to two or three dimensions while preserving the topology of the data. The 15 X 500 feature matrix obtained for each trial was resized to a 1D vector of 7500 parameters. The angular and force data were each normalized separately for all the subjects combined to a mean of zero and a unit standard deviation, thus resulting in a dimensionless vector. The perplexity parameters used ranged from 10 to 15 without substantial differences as suggested in [35].

#### kNN clustering and cross-validation

A weighted k-Nearest Neighbors (kNN) algorithm [36] was used to obtain a numerical value for the quality of clustering. The basic kNN algorithm classifies using a majority vote of the k nearest points to a given data point. The weighted algorithm gives each of the votes a specific weight. The weight we used was a squared inverse distance ($w_{i}=d_{i}^{-2}$ where *d*_*i*_ is the distance between the test point and the ith neighbor, *i* = 1 … *k*). For all our tests we used k = 10 nearest neighbors. The results were then cross-validated using k-fold cross-validation [37]. k-fold cross-validation means that the training data is divided to k bins where in every iteration a different bin is used as a test set, while the others are considered as the training set. The results for each of the folds are then averaged to gain the value that represents the quality of the classification. For all our tests we used k = 5 folds.

#### Sensitivity analysis

A sensitivity analysis algorithm was implemented in order to find the most distinctive features. Sensitivity analysis is a process in which the model output is tested under different input values [38]. In our case, the model output is the quality measure of the kNN clustering mentioned earlier, while the input parameters were each one of the feature vectors (finger forces and angles). It is important to note that we were not interested in the effects of each data point in the vector but rather each full feature vector.

The algorithm was designed to loop over the entire set of permutations possible over the set of features, for any number of chosen features in the range—[2, 14] (overall 32,751 permutations for each object). On each iteration, the features that were not chosen were replaced by a Gaussian noise N(0,1) (same as the data after normalization). Then the pipeline of t-SNE, kNN and cross-validation was used again. The parameters of this pipeline were not changed, since the number of initial dimensions and the distributions did not change. In view of the fact that t-SNE is a not a deterministic method (i.e. results are not identical over iterations), the process was repeated three times and the results were averaged for added robustness. At the end of this process, we had a set of 32,571 values (for each object), representing the quality of clustering for each of the different sets of chosen features. These five vectors were averaged to obtain a vector representing all the objects.

#### Analysis steps

The steps of the data analysis were as follows:

1. To examine if grasping patterns are subject-specific and, since grasping behavior varies greatly for different objects [15], data clustering was performed for each object separately. The clustering was based on all features to consider the effects on the patterns of both geometric properties exhibited mainly by joint angles and mechanical properties (weight, stiffness and friction), exhibited mainly by reaction forces.
2. To examine the effects of mechanical properties (weight variations in the bottles, stiffness and friction in the ellipsoid ball) on the clustering, we did the following:
  - Data clustering for each object separately based on angular data alone
  - Data clustering for the two bottles and the two ellipsoid balls using angular data alone and all the data collected
3. To find the most distinctive features, sensitivity analysis was performed for each object separately and all together based on all the features.

**Fig 5:**
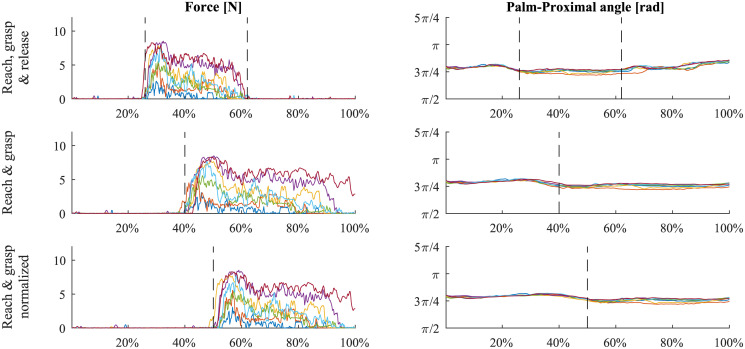
The three different time cropping and normalizations. Top row shows an example of the thumb reaction force (left) and one of the angles (right) using the entire sequence of data. Second row illustrates the same data but cropped after the object was released. The third is based on the same data but the reach and grasp are normalized to 250 points each to gain unity over the entire population.

All of these data analysis steps were conducted on the entire time series of 500 points (as described in the previous section). When discussing grasping tasks, all phases (reach, grasp and release) are relevant for analyzing the data. From a haptic feedback device perspective, the grasp phase is undoubtedly the most significant phase. The reach phase is also significant when attempting to predict the initial grasp parameters since the motor sequence that results in the object grasp initiates before the actual contact [39]. Bearing that in mind, we performed the same clustering process on the data without the release phase and with the reach and grasp phases normalized to the same length (see Fig 5, second and third rows). In this step, the forces were not considered during non-contact phases and were replaced by a Gaussian noise N(0,1).

Furthermore, in order to verify that the data collected was not time-dependent, 10% of the subjects were re-examined at a later time and their new data was added and classified along with their data collected in the first session. We hypothesized and found that the two sets of data were associated to the same cluster. This analysis was more qualitative in nature and was based on visually identifying the clusters.

## Results

### Grasping patterns are subject-specific

**Fig 6:**
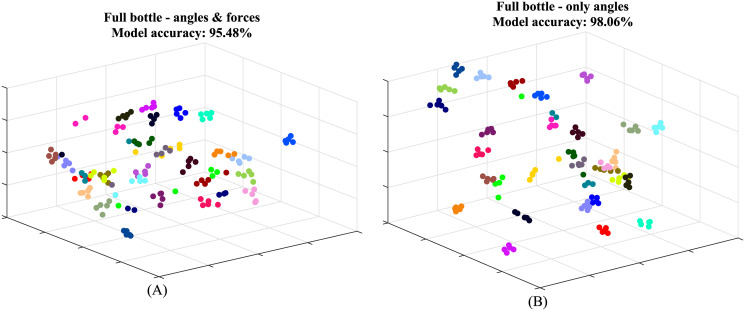
3D visualization of grasping data for the full water bottle. Each data point represents a specific trial and each subject is represented by a different color. The visualization was created based on (A) all the data collected; and (B) angular data alone.

As mentioned, all the features were used in this part of the analysis and each object was considered separately. The 3D visualization of the data generated by the t-SNE for the full bottle is illustrated in Fig 6A. Each data point represents a specific trial and each subject is represented by a different color; the colors were chosen to be as distinct as possible. While the visualization provides good evidence for the presence of distinct clusters, we calculated the cross validation of the kNN clustering for all the objects. The results are presented in the first row of Table 1; the average clustering success rate for all the objects is 95.48%.

**Table 1:** Cross-validation result for the weighted kNN clustering.

Data used	Visualization	Big ball	Full bottle	Half bottle	ellipsoid ball	ellipsoid plastic	Average
Angles & forces	Fig 6A	99.35%	95.48%	96.13%	96.77%	89.68%	95.48%
Only angles	Fig 6B	100%	98.06%	99.35%	98.71%	96.77%	98.58%
Only angles	Fig 7A		99.03%		98.39%		98.71%
Angles & forces	Fig 7B		97.10%		94.84%		95.97%
Angles & forces		98.05%	94.19%	94.84%	95.48%	87.26%	93.96%
Angles & forces		96.75%	93.55%	93.55%	90.32%	85.35%	91.90%
Angles & forces	Fig 10	97.63%					97.63%

First row contains the results for the main hypothesis using angles and forces. Second, third and fourth rows relate to the second part of our results; the second row contains the results of the clustering for each object separately using only angles. The third and fourth rows contain the results for the two pairs of identically shaped objects using only angles and using angles and forces respectively. The fifth and sixth rows contain the results for the different time normalizations, release cropped out and reach and grasp normalized to the same length, respectively. The seventh row shows the results of clustering different grasping types for the three geometrically different objects.

### Effects of mechanical properties on grasping patterns

**Fig 7:**
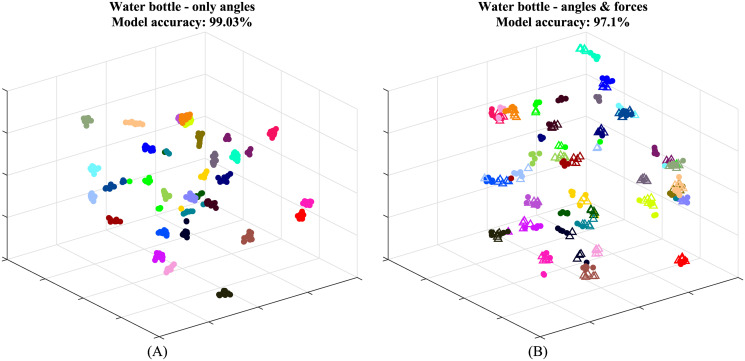
Visualization of the effects of mechanical properties—Weight variation. Each data point represents a specific trial and each subject is represented by a different color. (A) 3D visualization of angular data for the two bottles plotted on the same chart. (B) 3D visualization of all data collected for the two bottles, the full bottle is marked with a filled circle and the half-full bottle is marked with an empty triangle.

In the following section, we present the differences caused by changes of mechanical properties, weight variations in the bottles, stiffness and friction in the ellipsoid ball, while maintaining the same geometrical properties. First, we repeated the process of dimensionality reduction and clustering, using angular data alone. In this case, the feature vector contains 5000 data points. The visualization is presented in Fig 6B and the cross-validation results are presented in the second row of Table 1. The average success rate for all the objects is 98.58%. Next, to show that, upon considering angular data alone, the grasping behavior for geometrically identical pairs of objects is consistent, the angular data for the two bottles and the two ellipsoid balls was taken and passed through the same pipeline of t-SNE, kNN and cross-validation. The resulting visualization for the two bottles is shown in Fig 7A and the cross-validation results for both pairs of objects are laid out in the third row of Table 1 and are 98.39%-99.03%. Lastly, we tested clusters generated when considering both forces and angular data. The resulting visualization for the two bottles is shown in Fig 7B; the full bottle is marked with a filled circle and the half-full bottle is marked with an empty triangle. The quality value for both object pairs is in the fourth row of Table 1 and the values are 97.10%-94.84%.

### Which features are the most distinctive

**Fig 8:**
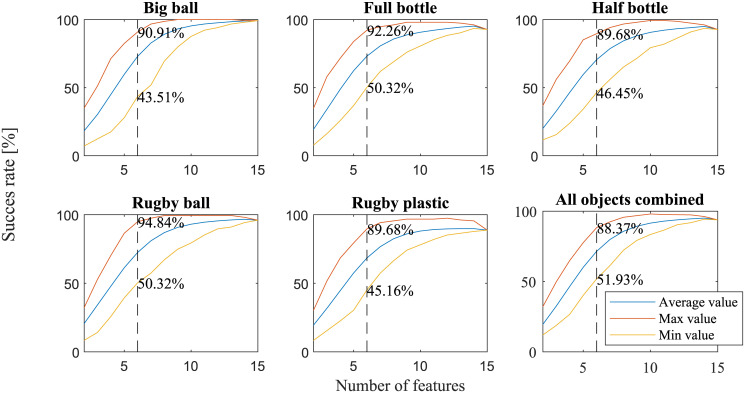
Quality of clustering Vs number of features. Graphs are shown for each of the objects and all of them combined (bottom right). The dotted line marks the six-feature value and the numbers represent the maximum and minimum values for six features.

The next part of our results was aimed at finding the most significant features for distinguishing between different subjects. In order to examine the effects of the number of features taken into account on the quality of clustering, the output of the sensitivity analysis was considered. For every number of features, we extracted the maximum, average and minimum quality value of the clustering. The results are shown in Fig 8.

It is apparent that the graphs start with a relative plateau, and the curve that represents the maximum value drops quickly when considering less than six features. At that point, the maximum success rate was ~90%, almost twice as much as the minimum success rate. For these reasons, we chose this point for our analysis and looked at the six features that provided the best result for each of the objects alone and all of them combined. These feature vectors are presented in Table 2. The columns represent each of the features and the first five rows each of the objects. The last row contains the results for all the objects combined. The chosen features are greyed out in the respective row and their success rate is shown in the last column.

**Table 2:** Best feature vectors and their respective success rates.

	Thumb			Index			Middle			Ring			Pinky			Success rate %
	*θ*_1_	*θ*_2_	*F*	*θ*_1_	*θ*_2_	*F*	*θ*_1_	*θ*_2_	*F*	*θ*_1_	*θ*_2_	*F*	*θ*_1_	*θ*_2_	*F*	
Full bottle																92.26%
Half bottle																89.68%
Tennis ball																90.91%
ellipsoid ball																94.84%
ellipsoid plastic																89.68%
All objects combined																88.37%

The table is used to illustrate the best six feature vectors (columns) for each of the objects and all of them combined (rows). The greyed cells represent the chosen features and the last columns represent the corresponding success rate.

### Effects of different time normalizations on the quality of clustering

The results of the different time normalizations are presented in the fifth and sixth rows of Table 1. When the release phase was removed, the average clustering quality decreased by 1.52% to 93.96%. A possible reason for the variance between individuals could potentially be the proportion between the duration of the reach and grasp phases. In order check this assumption, the reach and grasp phases were normalized to 250 data points each. The value further decreased by an additional 2.06% to an average clustering quality of 91.90%.

## Discussion

### Grasping patterns are subject-specific

The graph shown in Fig 6A is generated using all the features extracted, meaning two angle vectors and one force vector for each finger. The graph provides a very expressive illustration of the differences in individual grasping patterns. We can see 31 dense clusters, each comprised of around five trials. The resulting cross-validation values are considerably high (average = 95.48%), even without considering methods for removing outliers from the data. These values justify our main hypothesis and suggest that grasping patterns are indeed subject-specific.

**Fig 9:**
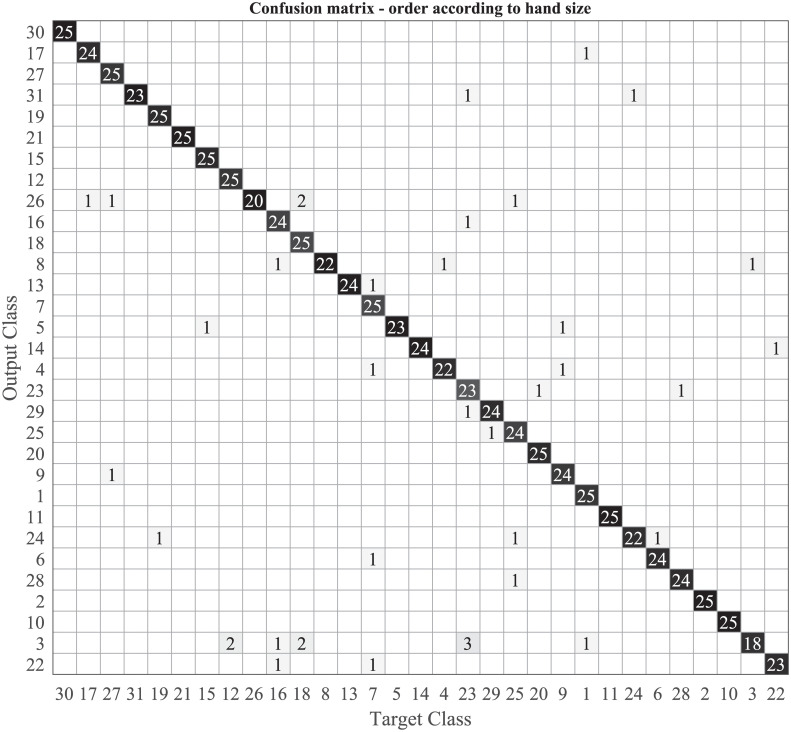
A Confusion matrix between all subjects across all objects, the matrix is arranged according to hand size.

This paper is mainly focused on showing that grasping patterns are subject specific and not on the exact factors that cause these differences. However, since the objects were the same while the subjects had different hand sizes, it was important to verify that a simple scaling of the objects would not cause the differences to disappear. To show that this is not the case, we can look at the confusion matrix between all the subjects shown in Fig 9. The order of the matrix is displayed according to hand size, defined as the distance from the wrist joint to the tip of the middle finger. It is apparent that there is a definite confusion between subjects with similar hand sizes (as indicated by the wrong outputs close to the main diagonal), but we also get wrong classifications for subjects with very different hand sizes. This implies that the hand size is not the only parameter that causes the difference.

### Effects of mechanical properties on grasping patterns

When considering angles alone, the values are higher than the ones obtained using forces in addition to angles. While a minor difference is acceptable, since the number of original dimensions was decreased from 7500 to 5000, the difference for some of the objects is higher than expected. We believe that this is caused by the noise seen in the force data. The motion capture system is very accurate (0.1mm error) in comparison to the force sensors. In addition, the low friction coefficient between the objects and the force sensors in our setup can cause some slippage of the objects and, in turn, vibrations that add noise to the force measurements. Although we were able to classify without the force vectors, we still believe that these vectors provide valuable data that relate to mechanical properties, mainly weight and stiffness and should not be omitted. This also relates to previous findings that the control of hand kinematics during grasping is separate from the control of reaction forces with the object [22].

When considering the angular data for the two pairs of geometrically identical objects (Fig 7A—the two bottles), we can see clusters of ~10 trials each for each of the subjects with high success rates. These results suggest that, upon alteration of the mechanical properties (weight variations in the bottles, stiffness and friction in the ellipsoid ball), the geometric grasping pattern does not change. When the force data was added (Fig 7B—the two bottles), each of the subject clusters was roughly divided into two similar and distinct clusters. This is caused by the difference in force caused by the difference in object weight. These results suggest that the mechanical properties of the object have an effect on the individual grasping pattern. Since the two individual clusters are very close, these effects are not as substantial as the differences between individuals. In order to validate this conclusion, further investigation with a larger set of objects is needed.

### Which features are the most distinctive

When looking at Fig 8, one can see that all the graphs demonstrate a similar behavior, and the success rate is ~90% for six features. When the number of features is above six, we can see that the graph plateaus, while for less than that, we get a very steep decline in the clustering quality. From the fact that at this point the maximum success rate value is almost twice as much as the minimum success rate, we can see that the different sets of features have a great significance on the results. While the setup, the success measure and the collected features are all different, the study conducted in [22] regarding grasp synergies shows a similar behavior, where information about the objects grasped increased with the increase of considered principal components, up to five-six principal components.

One can see from Table 2 that, overall, the thumb is the most significant finger for differentiation. This result could have been anticipated since the thumb has the most DoF and usually counteracts the forces applied on the object by the other fingers. Thus, the thumb does a more complex set of actions (kinematically and kinetically), leaving more room for subject differentiation. When considering heavier objects, such as the full bottle, the force vectors have a more significant role in the clustering process. It seems that the first angle of the index finger is more significant than the second one. For the middle finger, the relation is flipped. For the spherical and ellipsoid objects that are usually gripped using a “power sphere” grip [15], we can see that the second angle of the pinky is important; this is assumed to be caused by the extra movement of the pinky in relation to the other fingers. It is important to note that it is possible that some of the specific features found to be the most distinctive could potentially be replaced by a synergy of multiple features similar to findings shown in multiple studies in the past [22–24].

This could suggest that, according to the known grip type used for each object and its mechanical properties (mainly weight and stiffness), one can estimate which features would provide the best differentiation. Looking at the last row representing all the objects combined, we can see that, overall, the thumb and index finger are the most significant for distinction (representing four of the six chosen features); this could also have been anticipated since most known grip types are based on these two fingers [15].

### Effects of different time normalizations on the quality of clustering

As was mentioned in the previous section, all the above-mentioned data analysis was performed on the entire set of different phases since they are all part of the kinematic pattern of the grasp. Since our interest is haptics, we wanted to ensure that the release phase is not vital for the clustering. We can see from the results that the clustering quality decreased only by ~1.5%, which shows that the grasping patterns remain unique when considering only the first two phases and when not considering forces during non-contact motion. In addition, sensitivity analysis over different time windows (50 data points wide) shows that the most significant time windows for the clustering are around the grasp instance (end of reach beginning of grasp).

The clustering quality decreased by another ~2% when the reach and grasp were normalized to the same length. This suggests that the proportion between the durations contains some variance but is not very significant. This result is somewhat unsurprising, since it is known that the kinematic grasping patterns of humans are continuous and the proportion is relatively constant [39].

### Grasping patterns are object dependent

**Fig 10:**
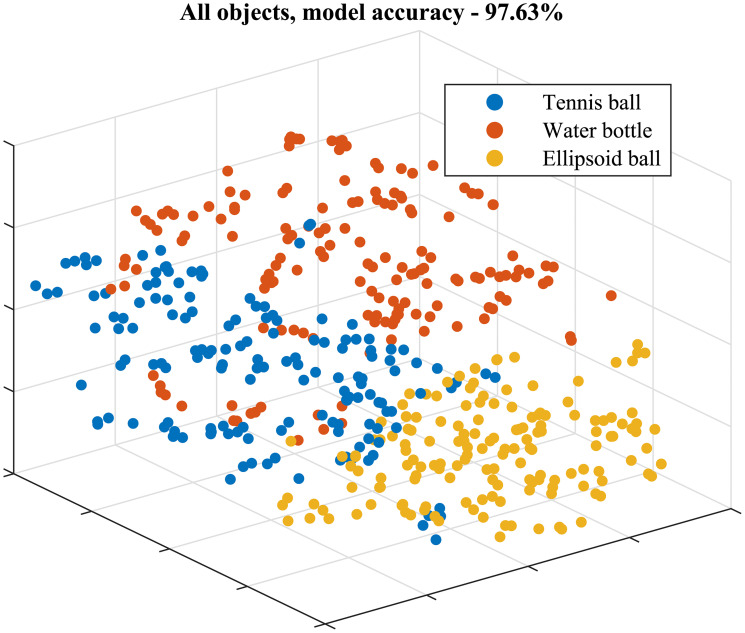
3D visualization of all the trials for the three geometrically different objects.

As mentioned earlier, previous research done on grasping behavior focused on finding common human grasping types [15]. To demonstrate that our data shows the same macro behavior, all the trials for the three geometrically different objects were taken together (rather than each object separately) into our clustering pipeline. In this case, to get a smoother result, the perplexity value used was 50 (within the range suggested in [35]). The results are shown in Fig 10. It is easy to see three distinct clusters for three different grasping behaviors. The cross-validation result of this clustering scheme is 98.9% (shown in the seventh row of Table 1). The results show that the data we collected contains both the macro behavior and the micro behavior of human grasping, thus further validating our results.

### Study limitations

While we measured forces at five points on the hand, it is obvious that these are not the only reaction forces between the hand and the object. We chose these points in view of our end goal, which was to design a haptic feedback device. These devices are usually limited in the number of points at which they can extract forces. We designed the experimental setup in view of the DoF that the device will have in the future and which are commonly used in existing designs.

As mentioned, the results were obtained based on five different objects varying in geometrical shape and mechanical properties (weight variations in the bottles, stiffness and friction in the ellipsoid ball). The results regarding these objects suggest interesting insights but further experiments with a wider range of objects are needed to validate these insights. Adding small objects that are gripped with a pinch or tripod grips to the set could be of interest.

## Conclusions

In this paper, we have shown that grasping patterns are subject-specific when considering reaction forces and joint angles and when considering joint angles alone. We also tested the effects of different mechanical properties, such as weight and stiffness on the classification, and found that the mechanical properties of the object have an effect on the individual grasping pattern. These effects are not as substantial as the differences between individuals. The reason for these differences is yet to be determined. We believe that there is a wide range of reasons, such as hand size, different finger lengths, hand strength, subjective preferences, etc. The collected data was then passed through a sensitivity analysis algorithm in order to find the most distinguishable features of the grasping pattern. We found that, for different sets comprised of the same number of features, we obtained a difference of roughly 45% in the clustering success rate. The resulting best features suggest a relation to known grip types and to previous studies on grasp synergies, but further investigation is needed. Lastly, the collected data was validated using the different objects and shown known behavior in classifying different grip types.

From the results shown and when considering that devices suitable for AR and VR are still limited in their capabilities (mainly due to lack of DoF), one could suggest that a smarter distribution of these DoF (both passive and active) could improve the quality of the output. These DoF affect both the tracking of the hand motion and the output of the system. As mentioned, current haptic feedback exoskeletons usually have only one DoF for each finger, including the thumb, even though its movement is very complex. Moreover, a learning control algorithm that adapts the output according to the user’s grasping behavior could further improve feedback quality. By designing a prediction model based on a deep recurrent neural network [40], our system will learn a model to predict future control values, thus reducing the control burden. The model will be trained using the entire population and will also be adjusted using a calibration scheme to each specific user, allowing for more accurate prediction. This approach is highly useful for us since we are planning to use soft robotic components which in many cases suffer from relatively long time constants and non-linear actuation profiles [41]. Lastly, the results reported extend the knowledge on human grasping, and the data analysis process used here could potentially be used in different research applications. Furthermore, the data collected offers a valuable dataset of human grasping behavior containing both forces and angular data.
